# Statement of Retraction: microRNA-10a-5p from gastric cancer cell-derived exosomes enhances viability and migration of human umbilical vein endothelial cells by targeting zinc finger MYND-type containing 11

**DOI:** 10.1080/21655979.2024.2299590

**Published:** 2024-02-20

We, the Editors and Publisher of the journal *Bioengineered*, have retracted the following article:

Jiaxin Zhu, Shasha Du, Jianfeng Zhang, Guangzhao Huang, Lujia Dong, Enbo Ren & Dechun Liu. (2022) microRNA-10a-5p from gastric cancer cell-derived exosomes enhances viability and migration of human umbilical vein endothelial cells by targeting zinc finger MYND-type containing 11, *Bioengineered* 13(1), doi: 10.1080/21655979.2021.2009962.

Since publication, significant concerns have been raised about the integrity of the data and reported results in the article. When approached for an explanation, the authors did not provide their original data or any necessary supporting information. As verifying the validity of published work is core to the integrity of the scholarly record, we are therefore retracting the article. All authors listed in this publication have been informed.

We have been informed in our decision-making by our editorial policies and the COPE guidelines.

The retracted article will remain online to maintain the scholarly record, but it will be digitally watermarked on each page as ‘Retracted’.

